# 2-[(*E*)-4-(Diethyl­amino)­styr­yl]-1-methyl­pyridinium iodide

**DOI:** 10.1107/S1600536810037505

**Published:** 2010-09-25

**Authors:** Narissara Kaewmanee, Kullapa Chanawanno, Suchada Chantrapromma, Hoong-Kun Fun

**Affiliations:** a Crystal Materials Research Unit, Department of Chemistry, Faculty of Science, Prince of Songkla University, Hat-Yai, Songkhla 90112, Thailand; b X-ray Crystallography Unit, School of Physics, Universiti Sains Malaysia, 11800 USM, Penang, Malaysia

## Abstract

In the title compound, C_18_H_23_N_2_^+^·I^−^, the cation exists in the *E* configuration with respect to the ethenyl C=C bond. The pyridinium and benzene rings are nearly coplanar, making a dihedral angle of 4.63 (7)°. The two ethyl groups of the diethyl­amino substituent point in opposite directions with respect to the benzene plane. In the crystal, the cation and the iodide anion are linked by a weak C—H⋯I inter­action. The cations are stacked in an anti-parallel manner along the *a* axis by a π–π inter­action with a centroid–centroid distance of 3.5262 (9) Å. The crystal structure is further stabilized by C—H⋯π inter­actions.

## Related literature

For bond-length data, see: Allen *et al.* (1987 ▶). For background to styryl pyridinium quaternary ammonium compounds, see: Browning *et al.* (1922 ▶, 1923 ▶); Chanawanno *et al.* (2010 ▶); Wainwright & Kristiansen (2003 ▶). For related structures, see: Chanawanno *et al.* (2008 ▶); Fun *et al.* (2009 ▶). For the stability of the temperature controller used in the data collection, see: Cosier & Glazer (1986 ▶).
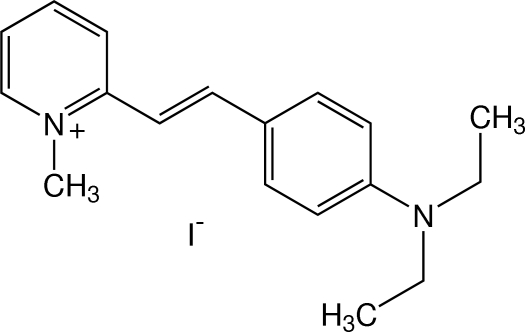


## Experimental

### 

#### Crystal data

C_18_H_23_N_2_^+^·I^−^
*M* *_r_* = 394.28
Monoclinic, 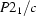

*a* = 7.7099 (1) Å
*b* = 20.2780 (4) Å
*c* = 10.9375 (2) Å
β = 92.527 (1)°
*V* = 1708.32 (5) Å^3^
*Z* = 4
Mo *K*α radiation
μ = 1.87 mm^−1^
*T* = 100 K
0.34 × 0.30 × 0.21 mm

#### Data collection

Bruker APEXII CCD area detector diffractometer
Absorption correction: multi-scan (*SADABS*; Bruker, 2005 ▶) *T*_min_ = 0.570, *T*_max_ = 0.691
23707 measured reflections
6198 independent reflections
5765 reflections with *I* > 2σ(*I*)
*R*_int_ = 0.026

#### Refinement

*R*[*F* ^2^ > 2σ(*F* ^2^)] = 0.022
*wR*(*F* ^2^) = 0.070
*S* = 1.10
6198 reflections
282 parameters
All H-atom parameters refined
Δρ_max_ = 0.51 e Å^−3^
Δρ_min_ = −0.63 e Å^−3^

### 

Data collection: *APEX2* (Bruker, 2005 ▶); cell refinement: *SAINT* (Bruker, 2005 ▶); data reduction: *SAINT*; program(s) used to solve structure: *SHELXTL* (Sheldrick, 2008 ▶); program(s) used to refine structure: *SHELXTL*; molecular graphics: *SHELXTL*; software used to prepare material for publication: *SHELXTL* and *PLATON* (Spek, 2009 ▶).

## Supplementary Material

Crystal structure: contains datablocks global, I. DOI: 10.1107/S1600536810037505/is2599sup1.cif

Structure factors: contains datablocks I. DOI: 10.1107/S1600536810037505/is2599Isup2.hkl

Additional supplementary materials:  crystallographic information; 3D view; checkCIF report

## Figures and Tables

**Table 1:** Hydrogen-bond geometry (Å, °) *Cg*2 is the centroid of the C8–C13 ring.

*D*—H⋯*A*	*D*—H	H⋯*A*	*D*⋯*A*	*D*—H⋯*A*
C1—H1*A*⋯I1^i^	0.91 (3)	2.99 (3)	3.7980 (18)	148.8 (19)
C18—H18*B*⋯*Cg*2^ii^	0.94 (3)	2.79 (3)	3.6270 (17)	149 (3)

Symmetry codes: (i) 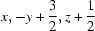; (ii) 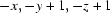.

**Fig. 1.**
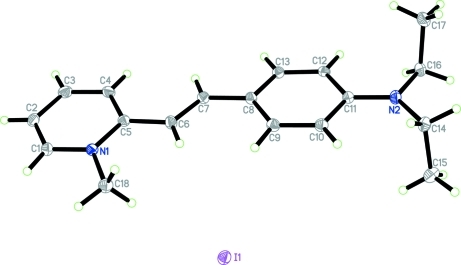
The molecular structure of the title compound, with 50% probability displacement ellipsoids and the atom-numbering scheme.

**Fig. 2.**
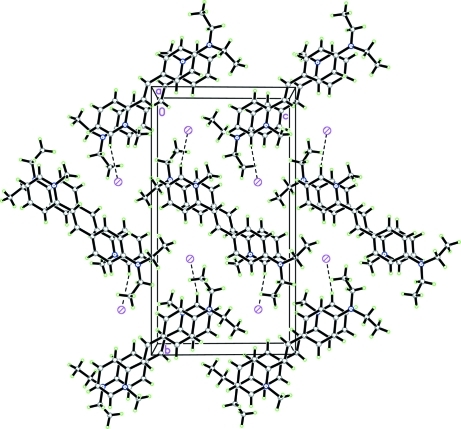
The crystal packing of the title compound viewed down the a axis. Weak C—H···I interactions are shown as dashed lines.

## supplementary crystallographic information

### Comment

For a long time, styryl pyridinium quaternary ammonium compounds were known to exhibit antiseptic properties (Browning *et al.*, 1922, 1923). However medicinal researchers have long neglected to further develop the styryl pyridinium chromophore compounds for use as antibacterial agents due to the superior properties of penicillin until the incoming of the penicillin-resistant bacteria phenomenon, for example, methicillin-resistant *Staphylococcus aureus*, MRSA. The most interesting feature of styryl pyridinium quaternary ammonium compounds is their very specific activity to MRSA which is a vital drug-resistant bacteria (Wainwright & Kristiansen, 2003; Chanawanno *et al.*, 2010). From this significant reason, our research group has synthesized and characterized several styryl pyridinium derivatives including the title compound (I) in order to search for new potent antibacterial agents. Herein we report the crystal structure of (I).

Figure 1 shows the asymmetric unit of (I), which consists of a C_18_H_23_N_2_^+^ cation and an I^-^ anion. The cation exists in the *E* configuration with respect to the C6═C7 double bond [1.350 (2) Å] with the torsion angle C5–C6–C7–C8 = -179.29 (16)°. The pyridinium and benzene rings are nearly coplanar with the ethenyl bridge with the dihedral angle between the pyridinium and benzene rings being 4.63 (7)°. The two ethyl groups of the diethylamino substituent pointed towards the opposite directions with respect to the plane of benzene ring. The conformation of the diethylamino can be indicated by the torsion angles C11–N2–C14–C15 = 84.7 (2)° and C11–N2–C16–C17 = 79.0 (2)°. The bond lengths of cation in (I) are in normal ranges (Allen *et al.*, 1987) and comparable to those in related structures (Chanawanno *et al.*, 2008; Fun *et al.*, 2009).

In the crystal packing (Fig. 2), the cations are arranged in a zig-zag manner along the *b* axis with the iodide ions located in the interstitials of the cations and linked to the cations by a C—H···I weak interaction (Table 1). The cations stacked approximately along the *a* axis in an antiparallel manner by π–π interaction with the *Cg*1···*Cg*2^iii^ distance of 3.5262 (9) Å [symmetry code: (iii) 1-x, 1-y, 1-z]; *Cg*1 and *Cg*2 are centroids of N1/C1–C5 and C8–C13 rings, respectively. The crystal structure is further stabilized by C—H···π interactions (Table 1).

### Experimental

The title compound (I) was prepared by mixing 1:1:1 molar ratio solutions of 1,2-dimethylpyridinium iodide (2 g, 8.5 mmol), 4-diethylaminobenzaldehyde (1.52 ml, 8.5 mmol) and piperidine (0.84 ml, 8.5 mmol) in methanol (40 ml). The resulting solution was refluxed for 6 hours under a nitrogen atmosphere. The orange solid which formed was filtered and washed with diethylether. Orange block-shaped single crystals of (I) suitable for *x*-ray structure determination were recrystallized from methanol by slow evaporation at room temperature over a few weeks (m.p. 527-529 K).

### Refinement

All H atoms were located in a difference map and refined isotropically. The highest residual electron density peak is located at 1.57 Å from I1 and the deepest hole is located at 0.48 Å from I1.

### Crystal data

**Table:**

C_18_H_23_N_2_^+^·I^−^	*F*(000) = 792
*M**_r_* = 394.28	*D*_x_ = 1.533 Mg m^−^^3^
Monoclinic, *P*2_1_/*c*	Melting point = 527–529 K
Hall symbol: -P 2ybc	Mo *K*α radiation, λ = 0.71073 Å
*a* = 7.7099 (1) Å	Cell parameters from 6198 reflections
*b* = 20.2780 (4) Å	θ = 2.0–32.6°
*c* = 10.9375 (2) Å	µ = 1.87 mm^−^^1^
β = 92.527 (1)°	*T* = 100 K
*V* = 1708.32 (5) Å^3^	Block, orange
*Z* = 4	0.34 × 0.30 × 0.21 mm

### Data collection

**Table:**

Bruker APEXII CCD area detector diffractometer	6198 independent reflections
Radiation source: sealed tube	5765 reflections with *I* > 2σ(*I*)
graphite	*R*_int_ = 0.026
φ and ω scans	θ_max_ = 32.6°, θ_min_ = 2.0°
Absorption correction: multi-scan (*SADABS*; Bruker, 2005)	*h* = −8→11
*T*_min_ = 0.570, *T*_max_ = 0.691	*k* = −29→30
23707 measured reflections	*l* = −16→16

### Refinement

**Table:**

Refinement on *F*^2^	Primary atom site location: structure-invariant direct methods
Least-squares matrix: full	Secondary atom site location: difference Fourier map
*R*[*F*^2^ > 2σ(*F*^2^)] = 0.022	Hydrogen site location: inferred from neighbouring sites
*wR*(*F*^2^) = 0.070	All H-atom parameters refined
*S* = 1.10	*w* = 1/[σ^2^(*F*_o_^2^) + (0.0312*P*)^2^ + 1.8072*P*] where *P* = (*F*_o_^2^ + 2*F*_c_^2^)/3
6198 reflections	(Δ/σ)_max_ = 0.001
282 parameters	Δρ_max_ = 0.51 e Å^−^^3^
0 restraints	Δρ_min_ = −0.63 e Å^−^^3^

### Special details

**Table:**

Experimental. The crystal was placed in the cold stream of an Oxford Cryosystems Cobra open-flow nitrogen cryostat (Cosier & Glazer, 1986) operating at 100.0 (1) K.
Geometry. All esds (except the esd in the dihedral angle between two l.s. planes) are estimated using the full covariance matrix. The cell esds are taken into account individually in the estimation of esds in distances, angles and torsion angles; correlations between esds in cell parameters are only used when they are defined by crystal symmetry. An approximate (isotropic) treatment of cell esds is used for estimating esds involving l.s. planes.
Refinement. Refinement of F^2^ against ALL reflections. The weighted R-factor wR and goodness of fit S are based on F^2^, conventional R-factors R are based on F, with F set to zero for negative F^2^. The threshold expression of F^2^ > 2sigma(F^2^) is used only for calculating R-factors(gt) etc. and is not relevant to the choice of reflections for refinement. R-factors based on F^2^ are statistically about twice as large as those based on F, and R- factors based on ALL data will be even larger.

### Fractional atomic coordinates and isotropic or equivalent isotropic displacement parameters (Å^2^)

**Table:**

	*x*	*y*	*z*	*U*_iso_*/*U*_eq_	
I1	0.385998 (14)	0.649927 (5)	0.254076 (9)	0.01744 (4)	
N1	0.27082 (18)	0.63425 (7)	0.69740 (12)	0.0128 (2)	
N2	0.1456 (2)	0.33502 (7)	0.09996 (13)	0.0165 (3)	
C1	0.3117 (2)	0.66702 (9)	0.80338 (15)	0.0156 (3)	
C2	0.3971 (2)	0.63637 (9)	0.89989 (15)	0.0169 (3)	
C3	0.4439 (2)	0.57021 (9)	0.88598 (15)	0.0181 (3)	
C4	0.4023 (2)	0.53711 (8)	0.77893 (15)	0.0159 (3)	
C5	0.3120 (2)	0.56923 (8)	0.68124 (14)	0.0130 (3)	
C6	0.2616 (2)	0.53758 (8)	0.56679 (15)	0.0149 (3)	
C7	0.3027 (2)	0.47469 (8)	0.53980 (14)	0.0141 (3)	
C8	0.2575 (2)	0.44086 (8)	0.42673 (14)	0.0136 (3)	
C9	0.1595 (2)	0.46906 (8)	0.32844 (15)	0.0144 (3)	
C10	0.1223 (2)	0.43480 (8)	0.22166 (15)	0.0150 (3)	
C11	0.1815 (2)	0.36902 (8)	0.20590 (14)	0.0135 (3)	
C12	0.2802 (2)	0.34044 (8)	0.30475 (15)	0.0131 (3)	
C13	0.3169 (2)	0.37578 (8)	0.41093 (14)	0.0133 (3)	
C14	0.0388 (2)	0.36290 (9)	−0.00116 (15)	0.0173 (3)	
C15	0.1390 (3)	0.40606 (10)	−0.08716 (17)	0.0216 (3)	
C16	0.2067 (2)	0.26717 (8)	0.08518 (15)	0.0164 (3)	
C17	0.0948 (3)	0.21691 (9)	0.14881 (17)	0.0201 (3)	
C18	0.1817 (2)	0.67224 (9)	0.59821 (16)	0.0182 (3)	
H1A	0.285 (3)	0.7107 (13)	0.805 (2)	0.020 (6)*	
H2A	0.426 (4)	0.6591 (13)	0.971 (3)	0.029 (7)*	
H3A	0.512 (4)	0.5499 (14)	0.954 (3)	0.031 (7)*	
H4A	0.434 (3)	0.4942 (13)	0.774 (2)	0.019 (6)*	
H6A	0.198 (3)	0.5628 (12)	0.507 (2)	0.014 (5)*	
H7A	0.369 (3)	0.4489 (13)	0.598 (2)	0.020 (6)*	
H9A	0.120 (3)	0.5160 (13)	0.332 (2)	0.021 (6)*	
H10A	0.055 (4)	0.4541 (14)	0.158 (3)	0.027 (7)*	
H12A	0.325 (3)	0.2966 (13)	0.298 (2)	0.020 (6)*	
H14A	−0.057 (3)	0.3869 (13)	0.030 (2)	0.017 (6)*	
H13A	0.384 (3)	0.3571 (11)	0.476 (2)	0.015 (6)*	
H14B	−0.013 (3)	0.3265 (13)	−0.047 (2)	0.021 (6)*	
H15A	0.061 (4)	0.4195 (15)	−0.156 (3)	0.037 (8)*	
H15B	0.238 (4)	0.3824 (14)	−0.117 (2)	0.026 (7)*	
H15C	0.180 (4)	0.4425 (15)	−0.047 (3)	0.028 (7)*	
H16A	0.323 (3)	0.2637 (13)	0.115 (2)	0.022 (6)*	
H16B	0.202 (3)	0.2567 (13)	0.002 (2)	0.020 (6)*	
H17A	0.139 (4)	0.1730 (15)	0.135 (3)	0.029 (7)*	
H17B	0.098 (3)	0.2228 (14)	0.236 (2)	0.021 (6)*	
H17C	−0.022 (4)	0.2207 (14)	0.121 (2)	0.027 (7)*	
H18A	0.176 (4)	0.7175 (14)	0.623 (2)	0.027 (7)*	
H18B	0.069 (4)	0.6552 (14)	0.586 (3)	0.032 (8)*	
H18C	0.244 (4)	0.6682 (14)	0.525 (3)	0.023 (6)*	

### Atomic displacement parameters (Å^2^)

**Table:**

	*U*^11^	*U*^22^	*U*^33^	*U*^12^	*U*^13^	*U*^23^
I1	0.02091 (6)	0.01714 (6)	0.01425 (6)	0.00473 (4)	0.00049 (4)	0.00028 (3)
N1	0.0135 (6)	0.0121 (6)	0.0130 (5)	0.0000 (4)	0.0022 (4)	0.0001 (4)
N2	0.0239 (7)	0.0128 (6)	0.0126 (6)	0.0009 (5)	−0.0015 (5)	−0.0015 (5)
C1	0.0179 (7)	0.0143 (7)	0.0147 (6)	−0.0003 (5)	0.0036 (5)	−0.0022 (5)
C2	0.0183 (7)	0.0200 (7)	0.0126 (6)	−0.0052 (6)	0.0026 (5)	−0.0017 (5)
C3	0.0205 (7)	0.0187 (7)	0.0149 (6)	−0.0030 (6)	−0.0018 (5)	0.0035 (6)
C4	0.0196 (7)	0.0125 (7)	0.0154 (6)	−0.0013 (5)	−0.0016 (5)	0.0020 (5)
C5	0.0121 (6)	0.0129 (6)	0.0140 (6)	−0.0011 (5)	0.0019 (5)	−0.0003 (5)
C6	0.0161 (7)	0.0143 (7)	0.0142 (6)	0.0000 (5)	−0.0013 (5)	−0.0013 (5)
C7	0.0142 (6)	0.0140 (7)	0.0141 (6)	−0.0010 (5)	0.0006 (5)	−0.0007 (5)
C8	0.0144 (6)	0.0128 (6)	0.0136 (6)	0.0001 (5)	0.0013 (5)	−0.0011 (5)
C9	0.0143 (6)	0.0128 (6)	0.0160 (6)	0.0015 (5)	0.0014 (5)	−0.0003 (5)
C10	0.0168 (7)	0.0137 (7)	0.0146 (6)	0.0021 (5)	−0.0005 (5)	0.0014 (5)
C11	0.0150 (6)	0.0128 (6)	0.0127 (6)	−0.0012 (5)	0.0011 (5)	−0.0010 (5)
C12	0.0134 (6)	0.0122 (6)	0.0140 (6)	−0.0014 (5)	0.0016 (5)	0.0012 (5)
C13	0.0136 (6)	0.0126 (6)	0.0138 (6)	0.0009 (5)	0.0011 (5)	0.0003 (5)
C14	0.0184 (7)	0.0195 (7)	0.0137 (6)	−0.0006 (6)	−0.0029 (5)	−0.0005 (6)
C15	0.0229 (8)	0.0255 (9)	0.0166 (7)	0.0000 (7)	0.0018 (6)	0.0029 (6)
C16	0.0208 (7)	0.0134 (7)	0.0150 (6)	0.0005 (6)	0.0019 (5)	−0.0033 (5)
C17	0.0229 (8)	0.0172 (8)	0.0205 (7)	−0.0029 (6)	0.0025 (6)	−0.0042 (6)
C18	0.0213 (8)	0.0171 (7)	0.0161 (7)	0.0037 (6)	−0.0008 (6)	0.0014 (6)

### Geometric parameters (Å, °)

**Table:**

N1—C1	1.361 (2)	C9—H9A	1.00 (3)
N1—C5	1.369 (2)	C10—C11	1.423 (2)
N1—C18	1.475 (2)	C10—H10A	0.93 (3)
N2—C11	1.366 (2)	C11—C12	1.418 (2)
N2—C14	1.463 (2)	C12—C13	1.383 (2)
N2—C16	1.466 (2)	C12—H12A	0.96 (3)
C1—C2	1.368 (2)	C13—H13A	0.94 (2)
C1—H1A	0.91 (3)	C14—C15	1.521 (3)
C2—C3	1.399 (3)	C14—H14A	0.96 (3)
C2—H2A	0.92 (3)	C14—H14B	0.97 (3)
C3—C4	1.375 (2)	C15—H15A	0.99 (3)
C3—H3A	0.98 (3)	C15—H15B	0.97 (3)
C4—C5	1.409 (2)	C15—H15C	0.91 (3)
C4—H4A	0.91 (3)	C16—C17	1.523 (3)
C5—C6	1.445 (2)	C16—H16A	0.94 (3)
C6—C7	1.350 (2)	C16—H16B	0.94 (2)
C6—H6A	0.95 (2)	C17—H17A	0.97 (3)
C7—C8	1.443 (2)	C17—H17B	0.96 (2)
C7—H7A	0.95 (3)	C17—H17C	0.94 (3)
C8—C9	1.408 (2)	C18—H18A	0.96 (3)
C8—C13	1.410 (2)	C18—H18B	0.94 (3)
C9—C10	1.378 (2)	C18—H18C	0.95 (3)
			
C1—N1—C5	122.25 (14)	C12—C11—C10	117.06 (14)
C1—N1—C18	117.02 (14)	C13—C12—C11	120.75 (15)
C5—N1—C18	120.72 (14)	C13—C12—H12A	119.0 (15)
C11—N2—C14	122.16 (15)	C11—C12—H12A	120.2 (15)
C11—N2—C16	120.88 (14)	C12—C13—C8	122.16 (14)
C14—N2—C16	116.90 (13)	C12—C13—H13A	120.7 (15)
N1—C1—C2	121.39 (16)	C8—C13—H13A	117.1 (15)
N1—C1—H1A	116.3 (16)	N2—C14—C15	113.93 (15)
C2—C1—H1A	122.2 (16)	N2—C14—H14A	110.1 (15)
C1—C2—C3	117.98 (15)	C15—C14—H14A	110.4 (15)
C1—C2—H2A	120.7 (18)	N2—C14—H14B	107.8 (16)
C3—C2—H2A	121.3 (18)	C15—C14—H14B	109.0 (16)
C4—C3—C2	120.60 (15)	H14A—C14—H14B	105 (2)
C4—C3—H3A	122.5 (17)	C14—C15—H15A	108.9 (18)
C2—C3—H3A	116.9 (17)	C14—C15—H15B	110.5 (17)
C3—C4—C5	120.59 (16)	H15A—C15—H15B	110 (2)
C3—C4—H4A	118.0 (16)	C14—C15—H15C	110.0 (18)
C5—C4—H4A	121.4 (16)	H15A—C15—H15C	109 (2)
N1—C5—C4	117.18 (14)	H15B—C15—H15C	108 (2)
N1—C5—C6	118.99 (14)	N2—C16—C17	112.73 (15)
C4—C5—C6	123.83 (15)	N2—C16—H16A	109.7 (16)
C7—C6—C5	123.55 (15)	C17—C16—H16A	109.9 (16)
C7—C6—H6A	118.6 (15)	N2—C16—H16B	108.7 (16)
C5—C6—H6A	117.8 (15)	C17—C16—H16B	107.1 (16)
C6—C7—C8	125.91 (15)	H16A—C16—H16B	109 (2)
C6—C7—H7A	119.8 (15)	C16—C17—H17A	109.6 (17)
C8—C7—H7A	114.3 (15)	C16—C17—H17B	112.3 (16)
C9—C8—C13	116.92 (14)	H17A—C17—H17B	106 (2)
C9—C8—C7	124.21 (15)	C16—C17—H17C	110.5 (17)
C13—C8—C7	118.86 (14)	H17A—C17—H17C	111 (2)
C10—C9—C8	121.80 (15)	H17B—C17—H17C	107 (2)
C10—C9—H9A	117.3 (14)	N1—C18—H18A	108.7 (16)
C8—C9—H9A	120.8 (14)	N1—C18—H18B	107.8 (18)
C9—C10—C11	121.30 (15)	H18A—C18—H18B	110 (2)
C9—C10—H10A	120.4 (17)	N1—C18—H18C	109.5 (17)
C11—C10—H10A	118.3 (17)	H18A—C18—H18C	110 (2)
N2—C11—C12	121.47 (15)	H18B—C18—H18C	111 (3)
N2—C11—C10	121.47 (15)		
			
C5—N1—C1—C2	0.1 (2)	C7—C8—C9—C10	179.02 (16)
C18—N1—C1—C2	−178.88 (16)	C8—C9—C10—C11	0.0 (3)
N1—C1—C2—C3	1.0 (3)	C14—N2—C11—C12	177.77 (15)
C1—C2—C3—C4	−1.1 (3)	C16—N2—C11—C12	0.6 (2)
C2—C3—C4—C5	0.1 (3)	C14—N2—C11—C10	−2.6 (3)
C1—N1—C5—C4	−1.1 (2)	C16—N2—C11—C10	−179.73 (15)
C18—N1—C5—C4	177.87 (15)	C9—C10—C11—N2	−179.66 (16)
C1—N1—C5—C6	178.99 (15)	C9—C10—C11—C12	0.0 (2)
C18—N1—C5—C6	−2.1 (2)	N2—C11—C12—C13	179.33 (15)
C3—C4—C5—N1	1.0 (2)	C10—C11—C12—C13	−0.3 (2)
C3—C4—C5—C6	−179.10 (16)	C11—C12—C13—C8	0.6 (2)
N1—C5—C6—C7	176.94 (16)	C9—C8—C13—C12	−0.6 (2)
C4—C5—C6—C7	−3.0 (3)	C7—C8—C13—C12	−179.43 (15)
C5—C6—C7—C8	−179.29 (16)	C11—N2—C14—C15	84.7 (2)
C6—C7—C8—C9	−1.4 (3)	C16—N2—C14—C15	−98.03 (19)
C6—C7—C8—C13	177.33 (16)	C11—N2—C16—C17	79.0 (2)
C13—C8—C9—C10	0.3 (2)	C14—N2—C16—C17	−98.24 (18)

### Hydrogen-bond geometry (Å, °)

**Table:**

*Cg*2 is the centroid of the C8–C13 ring.

**Table:**

*D*—H···*A*	*D*—H	H···*A*	*D*···*A*	*D*—H···*A*
C1—H1A···I1^i^	0.91 (3)	2.99 (3)	3.7980 (18)	148.8 (19)
C18—H18B···Cg2^ii^	0.94 (3)	2.79 (3)	3.6270 (17)	149 (3)

Symmetry codes: (i) *x*, −*y*+3/2, *z*+1/2; (ii) −*x*, −*y*+1, −*z*+1.
